# Maximum likelihood estimates of two-locus recombination fractions under some natural inequality restrictions

**DOI:** 10.1186/1471-2156-9-1

**Published:** 2008-01-04

**Authors:** Ying Zhou, Ning-Zhong Shi, Wing-Kam Fung, Jianhua Guo

**Affiliations:** 1 Key Laboratory for Applied Statistics of MOE and School of Mathematics and Statistics, Northeast Normal University, Changchun 130024, P. R. China; 2 Department of Statistics and Actuarial Science, The University of Hong Kong, Hong Kong, P. R. China

## Abstract

**Background:**

The goal of linkage analysis is to determine the chromosomal location of the gene(s) for a trait of interest such as a common disease. Three-locus linkage analysis is an important case of multi-locus problems. Solutions can be found analytically for the case of triple backcross mating. However, in the present study of linkage analysis and gene mapping some natural inequality restrictions on parameters have not been considered sufficiently, when the maximum likelihood estimates (MLEs) of the two-locus recombination fractions are calculated.

**Results:**

In this paper, we present a study of estimating the two-locus recombination fractions for the phase-unknown triple backcross with two offspring in each family in the framework of some natural and necessary parameter restrictions. A restricted expectation-maximization (EM) algorithm, called REM is developed. We also consider some extensions in which the proposed REM can be taken as a unified method.

**Conclusion:**

Our simulation work suggests that the REM performs well in the estimation of recombination fractions and outperforms current method. We apply the proposed method to a published data set of mouse backcross families.

## Background

Molecular genetics has made much progress in recent years, among which linkage analysis fulfills an important role. Genetic linkage refers to the ordering of genetic loci on a chromosome and to estimating genetic distances among them, where these distances are determined on the basis of a statistical phenomenon. Statistical machinery has been used to analyze family data and to detect linkage [1-4]. The degree of linkage can be measured by recombination fraction. The proportion of recombinant haplotypes (or offspring) potentially produced by a doubly heterozygous parent is called recombination fraction, which is also the probability of occurrence of a recombination. Many map functions under different assumptions have been derived [5-7], from which the genetic distance and the recombination fraction can be mutually transformed. Human gene mapping is now an important field of science. A critical first step in finding gene loci that contribute to a genetic trait is to demonstrate linkage with a gene of known location (marker). So estimating the recombination fractions is important in linkage analysis.

In several respects, three-locus analysis yields more information than does two-locus analysis [8-11]. Three-locus linkage analysis is also an important case of multi-locus problems. Methods for detecting multilocus linkage in humans and estimation of recombination have been proposed by Lathrop *et al*. [12], and Lathrop [13]. More recently, Ott [3] has considered the estimation of two-locus recombination fractions for phase-unknown triple backcross families with two offspring in each family. The author gave the presentations of the estimates of the two-locus recombination fractions. Wu *et al*. [9] considered simultaneous estimation of linkage and linkage phases in outcrossing species. However, as mentioned in Ott [3], the estimates suggested by the author may not satisfy some natural restrictions which two-locus recombination fractions should satisfy in fact. One may not obtain a reasonable interpretation on the recombination phenomenon among loci based on the estimates. Furthermore, illegimate estimates of recombination fractions may also reduce the power to detect linkage which can provide irresponsible evidence to the researchers. In addition, the restrictions on recombination fractions given in the context are necessary in linkage analysis. For example, they can be applied to determine the locus order on the chromosome [9-11].

This estimation problem of two-locus recombination fractions in three-locus linkage analysis belongs to the constrained parameter problems which are not only important but also appear in many areas. The reader is referred to [14-17]. However, the methods provided in the literatures cannot be directly applied to the above genetics problem.

Motivated by this unsolved problem that the restrictions on recombination fractions have not been considered sufficiently, in this paper, we consider the estimation of the two-locus recombination fractions under some natural and necessary restrictions. We develop a restricted EM algorithm, called REM, which gives estimating results through taking account of the natural inequality restrictions on the two-locus recombination fractions, and the algorithm has been implemented by computer. Moreover, this algorithm can be easily generalized to other cases, and the REM performs well as a unified approach. Simulation studies show that our new method works well in each scenario and has advantages over current method, in other words, the major advantages of our method is its robustness and efficiency. An example is used to validate the application of our method to linkage analysis.

## Methods

Consider three biallele marker loci, where alleles are designed as *A*, *a*; *B*, *b*; *C*, *c *at loci A, B, C, respectively, with the order of loci being A-B-C. Assume a triply homozygous parent *abc*/*abc*, and a triply heterozygous parent (*A*/*a*, *B*/*b*, *C*/*c*). For the latter, there are four possible phases: (I) *ABC*/*abc*, (II) *ABc*/*abC*, (III) *AbC*/*aBc*, (IV) *Abc*/*aBC*. As Ott [3] pointed out, under regular conditions (linkage equilibrium), each of these phases occurs with probability 1/4. When it is not the case, we let the prior probability be *h*_*i *_(*i *= 1, 2, 3, 4) in a later section, and give corresponding feasible approach.

Each offspring only receives haplotype *abc *from the triply homozygous parent, but receives one of the eight possible kinds of haplotypes from the heterozygous parent, which can be seen at the second column of Table 1. The last four columns of Table 1 give the conditional probabilities with which the offspring phenotypes occur given the parental phase, and the first column presents the code for each haplotype that we will use. For the phase-unknown triple backcross, each haplotype symbol listed in Table 1 just corresponds to one offspring phenotype of the markers.

**Table 1:** Conditional haplotype probabilities given phase produced by a triply heterozygous parent

		Phase			
		
*i*	Haplotype	I	II	III	IV
1	ABC	*g*_00_/2	*g*_01_/2	*g*_11_/2	*g*_10_/2
2	*ABc*	*g*_01_/2	*g*_00_/2	*g*_10_/2	*g*_11_/2
3	*AbC*	*g*_11_/2	*g*_10_/2	*g*_00_/2	*g*_01_/2
4	*Abc*	*g*_10_/2	*g*_11_/2	*g*_01_/2	*g*_00_/2
5	*aBC*	*g*_10_/2	*g*_11_/2	*g*_01_/2	*g*_00_/2
6	*aBc*	*g*_11_/2	*g*_10_/2	*g*_00_/2	*g*_01_/2
7	*abC*	*g*_01_/2	*g*_00_/2	*g*_10_/2	*g*_11_/2
8	*abc*	*g*_00_/2	*g*_01_/2	*g*_11_/2	*g*_10_/2
		
Total		1	1	1	1

*g*_00_, *g*_01_, *g*_10 _and *g*_11 _denote joint recombination fractions, where the subscript 1 represents recombination, and 0 represents nonrecombination.

Let *θ*_*AB*_, *θ*_*BC *_and *θ*_*AC*_, respectively denote two-locus recombination fractions between loci A and B, between loci B and C, and between loci A and C; *g*_00_, *g*_01_, *g*_10 _and *g*_11 _denote joint recombination fractions, where the subscript 1 represents recombination, and 0 represents non-recombination, e.g., *g*_10 _is the probability of single recombinant with a recombination for loci A and B but none for loci B and C. So it is clear that the following equations hold:

*θ*_*AB *_= *g*_11 _+ *g*_10_, *θ*_*BC *_= *g*_11 _+ *g*_01_, *θ*_*AC *_= *g*_10 _+ *g*_01_.

Ott [3] groups all possible two-offspring haplotype pairs into four phenotype classes with probability *p*_*k *_(*k *= 1, 2, 3, 4) according to linkage analysis regulation. These classes are reproduced in Table 2, in which the second column represents two-offspring haplotype pairs, corresponding to two phenotypes. Taking (*i*, *j*) = (5, 6) as an example, we say one of the sib pair expresses phenotype *aa*/*Bb*/*Cc*, and the other expresses phenotype *aa*/*Bb*/*cc*. There is no order relationship between *i *and *j*. The probabilities of occurrence for all 8 × 9/2 = 36 possible pairs of offspring's phenotypes can be calculated easily, e.g., the joint probability of occurrence of phenotypes *aa*/*Bb*/*Cc *and *aa*/*Bb*/*cc *(diplotypes *aBC*/*abc *and *aBc*/*abc*) is (*g*_11_*g*_10 _+ *g*_01_*g*_00_)/4. It then turns out that, among the 36 probabilities, only four different values occur so that phenotypes with the same probabilities may be combined a single class and four classes are obtained.

**Table 2:** Phenotype classes for phase-unknown triple backcross families with two offspring

*k*	(*i*, *j*)^*a*^	*p*_*k*_
1	(1,1), (2,2), (3,3), (4,4), (5,5), (6,6) (7,7), (8,8), (4,5), (3,6), (2,7), (1,8)	$g_{11}^{2}+g_{10}^{2}+g_{01}^{2}+g_{00}^{2}$
2	(1,2), (3,4), (3,5), (1,7), (4,6), (2,8), (5,6), (7,8)	2(*g*_11_*g*_10 _+ *g*_01_*g*_00_)
3	(2,3), (1,4), (1,5), (2,6), (3,7), (4,8), (6,7), (5,8)	2(*g*_11_*g*_01 _+ *g*_10_*g*_00_)
4	(1,3), (2,4), (2,5), (1,6), (4,7), (3,8), (5,7), (6,8)	2(*g*_11_*g*_00 _+ *g*_10_*g*_01_)
		
Total		1

^*a*^(*i*, *j*): *i *and *j *refer to the code of haplotype in Table 1, corresponding to a phenotype each.

Let the total number of families (or sib pairs) observed be *n*, and the number of families which are grouped into class *k *be *n*_*k *_(*k *= 1, 2, 3, 4). Then (*n*_1_, *n*_2_, *n*_3_, *n*_4_) is multinomial distributed with probability (*p*_1_, *p*_2_, *p*_3_, *p*_4_), and $\sum_{k=1}^{4}n_{k}=n$. The MLEs of *p*_*k*_'s are ${\hat{p}}_{k}=\frac{n_{k}}{n}$ (*k *= 1, 2, 3, 4). Using the function relationships given in equations (1) and Table 2, as well as the property of MLE, the MLEs of *θ*_*AB*_, *θ*_*BC *_and *θ*_*AC *_can be obtained as Ott [3]. We call this method the unrestricted method that gives unrestricted estimates, and let ${\hat{\theta }}^{U}=\left({\hat{\theta }}_{AB}^{U},{\hat{\theta }}_{BC}^{U},{\hat{\theta }}_{AC}^{U}\right)$ denote the unrestricted MLE, where

$$\begin{array}{ll} {\hat{\theta }}_{AB}^{U}=\{\begin{array}{l} 1/2-1/2\sqrt{1-2({\hat{p}}_{3}+{\hat{p}}_{4})},\\ 1/2, \end{array} & \begin{array}{l} \text{if }{\hat{p}}_{3}+{\hat{p}}_{4}<1/2,\\ \text{otherwise,} \end{array}\\ {\hat{\theta }}_{BC}^{U}=\{\begin{array}{l} 1/2-1/2\sqrt{1-2({\hat{p}}_{2}+{\hat{p}}_{4})},\\ 1/2, \end{array} & \begin{array}{l} \text{if }{\hat{p}}_{2}+{\hat{p}}_{4}<1/2,\\ \text{otherwise,} \end{array}\\ {\hat{\theta }}_{AC}^{U}=\{\begin{array}{l} 1/2-1/2\sqrt{1-2({\hat{p}}_{2}+{\hat{p}}_{3})},\\ 1/2, \end{array} & \begin{array}{l} \text{if }{\hat{p}}_{2}+{\hat{p}}_{3}<1/2,\\ \text{otherwise}\text{.} \end{array} \end{array}$$

### Natural inequality restrictions on parameters

In parameter estimation, not only the data structure but also the restrictions on the parameters should be considered, otherwise the MLEs obtained may be unreasonable. For two-locus recombination fractions, the following inequality restrictions: *θ*_*AB *_≤ *θ*_*BC *_+ *θ*_*AC*_, *θ*_*BC *_≤ *θ*_*AB *_+ *θ*_*AC*_, *θ*_*AC *_≤ *θ*_*AB *_+ *θ*_*BC*_, and 0 ≤ *θ*_*AB*_, *θ*_*BC*_, *θ*_*AC *_≤ 1/2 must be considered. For the given order of loci A-B-C, additional restrictions: *θ*_*AB *_≤ *θ*_*AC *_and *θ*_*BC *_≤ *θ*_*AC *_are required. Combining all these inequalities, the following equivalent restrictions are obtained:

$$\{\begin{array}{l} \theta_{AB}\leq \theta_{AC},\\ \theta_{BC}\leq \theta_{AC},\\ \theta_{AC}\leq \theta_{AB}+\theta_{BC},\\ \theta_{AC}\leq 1/2. \end{array}$$

These restrictions are natural and necessary.

### Proposed algorithm

In this section, we propose an approach to calculate MLEs of two-locus recombination fractions under restriction (2), which works well in application. From equations (1) and Table 2, *p*_*k*_'s are functions of independent parameters *g*_10_, *g*_01 _and *g*_11_, and also functions of *θ*_*AB*_, *θ*_*BC *_and *θ*_*AC*_, so the log-likelihood function can be written as the following form

$$l(\theta |\{n_{k}\})=\sum_{k=1}^{4}n_{k}\ln(p_{k}(\theta_{AB},\theta_{BC},\theta_{AC})),$$

where *θ *= (*θ*_*AB*_, *θ*_*BC*_, *θ*_*AC*_). Our goal is to find ${\hat{\theta }}^{R}=\left({\hat{\theta }}_{AB}^{R},{\hat{\theta }}_{BC}^{R},{\hat{\theta }}_{AC}^{R}\right)$, such that $l({\hat{\theta }}^{R}|\{n_{k}\})=\underset{\theta }{\max}l(\theta |\{n_{k}\})$ under restriction (2), where ${\hat{\theta }}^{R}$ denotes the restricted MLE of *θ*.

We propose our restricted EM algorithm (REM) on the basis of the EM algorithm of Dempster et al. [18] as follows:

Augment the observed data {*n*_*k*_, *k *= 1, 2, 3, 4} by latent variables {*n*_*kl*_, *k*, *l *= 1, 2, 3, 4} to obtain a complete data set, where $n_{k}=\sum_{l=1}^{4}n_{kl}$, and {*n*_*kl*_, *k*, *l *= 1, 2, 3, 4} is multinomial distributed with probability {*p*_*kl*_, *k*, *l *= 1, 2, 3, 4}. Here, *p*_*kl *_are components of *p*_*k *_in Table 2 with $p_{11}=g_{00}^{2},p_{12}=g_{01}^{2},p_{13}=g_{10}^{2},p_{14}=g_{11}^{2}$; *p*_21 _= *g*_00_*g*_01_, *p*_22 _= *g*_00_*g*_01_, *p*_23 _= *g*_10_*g*_11_, *p*_24 _= *g*_10_*g*_11_; *p*_31 _= *g*_00_*g*_10_, *p*_32 _= *g*_00_*g*_10_, *p*_33 _= *g*_01_*g*_11_, *p*_34 _= *g*_01_*g*_11_; *p*_41 _= *g*_00_*g*_11_, *p*_42 _= *g*_00_*g*_11_, *p*_43 _= *g*_01_*g*_10_, *p*_44 _= *g*_01_*g*_10_. *n*_*kl *_have its interpretation, for example, *n*_11 _can be interpreted as the number of the families: (phase I → (1,1) or (8,8) or (1,8)), or (phase II → (2,2) or (7,7) or (2,7)), or (phase III → (4,4) or (5,5) or (4,5)), or (phase IV → (3,3) or (6,6) or (3,6)), where (phase I → (1,1)) denotes the event that the families have phase I, and the haplotype pairs of their offspring are (1,1), and other notations are analogous to interpret.

Because parameters *θ*_*AB*_, *θ*_*BC*_, and *θ*_*AC *_are equivalent to independent parameters *g*_10_, *g*_01 _and *g*_11_, we still consider parameters *g*_10_, *g*_01 _and *g*_11 _here, and restriction (2) is equivalent to the following restriction (3):

$$\{\begin{array}{l} g_{11}\leq g_{01},\\ g_{11}\leq g_{10},\\ g_{11}\geq 0,\\ g_{01}+g_{10}\leq 1/2. \end{array}$$

Thus, finding MLE ${\hat{g}}^{R}$ (the restricted MLE of **g **= (*g*_10_, *g*_01_, *g*_11_), such that $l({\hat{g}}^{R}|\{n_{k}\})=\underset{g}{\max}l(g|\{n_{k}\})$) under restriction (3) implies finding MLE ${\hat{\theta }}^{R}$ of *θ *under (2). The complete data log-likelihood function can be written as

$$\begin{array}{cc} l(g|\{n_{kl}\})=\sum_{k=1}^{4}\sum_{l=1}^{4}n_{kl}\ln(p_{kl}(g)), & k,l=1,2,3,4, \end{array}$$

where *p*_*kl*_'s are functions of **g **as given above. The conditional expectation of *l*(**g**|{*n*_*kl*_}) when the *s*th step parameter values **g**^(*s*) ^are given is

$$Q(g|g^{\left(s\right)},\{n_{k}\})=2[a_{1}^{\left(s+1\right)}ln(1-g_{01}-g_{10}-g_{11})+a_{2}^{\left(s+1\right)}ln(g_{01})+a_{3}^{\left(s+1\right)}ln(g_{10})+a_{4}^{\left(s+1\right)}ln(g_{11})],$$

where

$$\begin{array}{l} a_{1}^{\left(s+1\right)}=n_{1}\frac{{\left(g_{00}^{\left(s\right)}\right)}^{2}}{p_{1}^{\left(s\right)}}+n_{2}\frac{g_{00}^{\left(s\right)}g_{01}^{\left(s\right)}}{p_{2}^{\left(s\right)}}+n_{3}\frac{g_{00}^{\left(s\right)}g_{10}^{\left(s\right)}}{p_{3}^{\left(s\right)}}+n_{4}\frac{g_{00}^{\left(s\right)}g_{11}^{\left(s\right)}}{p_{4}^{\left(s\right)}},\\ a_{2}^{\left(s+1\right)}=n_{1}\frac{{\left(g_{01}^{\left(s\right)}\right)}^{2}}{p_{1}^{\left(s\right)}}+n_{2}\frac{g_{00}^{\left(s\right)}g_{01}^{\left(s\right)}}{p_{2}^{\left(s\right)}}+n_{3}\frac{g_{01}^{\left(s\right)}g_{11}^{\left(s\right)}}{p_{3}^{\left(s\right)}}+n_{4}\frac{g_{01}^{\left(s\right)}g_{10}^{\left(s\right)}}{p_{4}^{\left(s\right)}},\\ a_{3}^{\left(s+1\right)}=n_{1}\frac{{\left(g_{10}^{\left(s\right)}\right)}^{2}}{p_{1}^{\left(s\right)}}+n_{2}\frac{g_{11}^{\left(s\right)}g_{10}^{\left(s\right)}}{p_{2}^{\left(s\right)}}+n_{3}\frac{g_{00}^{\left(s\right)}g_{10}^{\left(s\right)}}{p_{3}^{\left(s\right)}}+n_{4}\frac{g_{01}^{\left(s\right)}g_{10}^{\left(s\right)}}{p_{4}^{\left(s\right)}},\\ a_{4}^{\left(s+1\right)}=n_{1}\frac{{\left(g_{11}^{\left(s\right)}\right)}^{2}}{p_{1}^{\left(s\right)}}+n_{2}\frac{g_{11}^{\left(s\right)}g_{10}^{\left(s\right)}}{p_{2}^{\left(s\right)}}+n_{3}\frac{g_{01}^{\left(s\right)}g_{11}^{\left(s\right)}}{p_{3}^{\left(s\right)}}+n_{4}\frac{g_{00}^{\left(s\right)}g_{11}^{\left(s\right)}}{p_{4}^{\left(s\right)}}. \end{array}$$

Then the restricted estimating problem may be written as

*Max Q*(**g**|**g**^(*s*)^, {*n*_*k*_}), subject to g satisfies restriction (3).

The Hessian matrix of *Q*(**g**|**g**^(*s*)^, {*n*_*k*_}) for *g*_10_, *g*_01 _and *g*_11 _is negative definite, so *Q*(**g**|**g**^(*s*)^, {*n*_*k*_}) is strictly concave for *g*_10_, *g*_01 _and *g*_11_. This implies that there exists one unique point ${\tilde{g}}^{\left(s+1\right)}=({\tilde{g}}_{10}^{\left(s+1\right)},{\tilde{g}}_{01}^{\left(s+1\right)},{\tilde{g}}_{11}^{\left(s+1\right)})$ satisfying $Q({\tilde{g}}^{\left(s+1\right)}|g^{\left(s\right)},\{n_{k}\})=\underset{g}{\max}Q(g|g^{\left(s\right)},\{n_{k}\})$. Following some calculation, it is easy to obtain that ${\tilde{g}}_{10}^{\left(s+1\right)}=a_{3}^{\left(s+1\right)}/n,{\tilde{g}}_{01}^{\left(s+1\right)}=a_{2}^{\left(s+1\right)}/n$ and ${\tilde{g}}_{11}^{\left(s+1\right)}=a_{4}^{\left(s+1\right)}/n$. If ${\tilde{g}}^{\left(s+1\right)}$ satisfies restriction (3), then $g^{\left(s+1\right)}={\tilde{g}}^{\left(s+1\right)}$ in the (*s *+ 1)th iteration for EM algorithm, otherwise, we use the Kuhn-Tucker conditions [19,20] to deal with problem (5). Thus, we can still find a unique point $ˇg^{\left(s+1\right)}=(ˇg_{10}^{\left(s+1\right)},ˇg_{01}^{\left(s+1\right)},ˇg_{11}^{\left(s+1\right)})$, such that $Q(ˇg^{\left(s+1\right)}|g^{\left(s\right)},\{n_{k}\})=\underset{g}{\max}Q(g|g^{\left(s\right)},\{n_{k}\})$ under restriction (3), because *Q*(**g**|**g**^(*s*)^, {*n*_*k*_}) is a strictly concave function for *g*_10_, *g*_01 _and *g*_11 _and the restriction region is a convex set. See Appendix for the Kuhn-Tucker conditions and the solving process of $ˇg^{\left(s+1\right)}$.

We give the complete REM algorithm as follows:

Let $g^{\left(0\right)}=(g_{10}^{\left(0\right)},g_{01}^{\left(0\right)},g_{11}^{\left(0\right)})$ be the starting point (the starting value of **g**^(0) ^may be taken as ${\hat{g}}^{U}=({\hat{g}}_{10}^{U},{\hat{g}}_{01}^{U},{\hat{g}}_{11}^{U})$ which can make the REM converge faster, where $\left({\hat{g}}_{10}^{U},{\hat{g}}_{01}^{U},{\hat{g}}_{11}^{U}\right)$ can be obtained from $\left({\hat{\theta }}_{AB}^{U},{\hat{\theta }}_{BC}^{U},{\hat{\theta }}_{AC}^{U}\right)$ by equations (1));

E-step: At step *s*, compute the expected number of recombination events $a^{\left(s+1\right)}=(a_{1}^{\left(s+1\right)},a_{2}^{\left(s+1\right)},a_{3}^{\left(s+1\right)},a_{4}^{\left(s+1\right)})$ from **g**^(*s*)^;

M-step: Compute **g**^(*s*+1) ^using **a**^(*s*+1)^. Firstly, compute ${\tilde{g}}^{\left(s+1\right)}=({\tilde{g}}_{10}^{\left(s+1\right)},{\tilde{g}}_{01}^{\left(s+1\right)},{\tilde{g}}_{11}^{\left(s+1\right)})=(a_{3}^{\left(s+1\right)}/n,a_{2}^{\left(s+1\right)}/n,a_{4}^{\left(s+1\right)}/n)$. If ${\tilde{g}}^{\left(s+1\right)}$ satisfies restriction (3), then **g**^(*s*+1) ^= ${\tilde{g}}^{\left(s+1\right)}$; otherwise, then **g**^(*s*+1) ^must belong to one of the following cases (i.e. only one case holds):

case 1. $g_{00}^{\left(s+1\right)}=g_{01}^{\left(s+1\right)}=g_{10}^{\left(s+1\right)}=g_{11}^{\left(s+1\right)}=1/4$, if the following inequalities hold simultaneously

$$\{\begin{array}{l} a_{3}^{\left(s+1\right)}+a_{4}^{\left(s+1\right)}>a_{1}^{\left(s+1\right)}+a_{2}^{\left(s+1\right)},\\ a_{2}^{\left(s+1\right)}+a_{4}^{\left(s+1\right)}>a_{1}^{\left(s+1\right)}+a_{3}^{\left(s+1\right)},\\ a_{2}^{\left(s+1\right)}+a_{3}^{\left(s+1\right)}+a_{4}^{\left(s+1\right)}>3a_{1}^{\left(s+1\right)}; \end{array}$$

case 2. $g_{01}^{\left(s+1\right)}=g_{11}^{\left(s+1\right)}=g_{10}^{\left(s+1\right)}=\frac{a_{2}^{\left(s+1\right)}+a_{3}^{\left(s+1\right)}+a_{4}^{\left(s+1\right)}}{3n}$, $g_{00}^{\left(s+1\right)}=\frac{a_{1}^{\left(s+1\right)}}{n}$, if

$$\{\begin{array}{l} a_{3}^{\left(s+1\right)}+a_{4}^{\left(s+1\right)}>2a_{2}^{\left(s+1\right)},\\ a_{2}^{\left(s+1\right)}+a_{4}^{\left(s+1\right)}>2a_{3}^{\left(s+1\right)},\\ 3n/4\geq a_{2}^{\left(s+1\right)}+a_{3}^{\left(s+1\right)}+a_{4}^{\left(s+1\right)}>0; \end{array}$$

case 3. $g_{01}^{\left(s+1\right)}=g_{11}^{\left(s+1\right)}=\frac{a_{2}^{\left(s+1\right)}+a_{4}^{\left(s+1\right)}}{2n}$, $g_{10}^{\left(s+1\right)}=g_{00}^{\left(s+1\right)}=\frac{a_{1}^{\left(s+1\right)}+a_{3}^{\left(s+1\right)}}{2n}$, if

$$\{\begin{array}{l} a_{3}^{\left(s+1\right)}a_{4}^{\left(s+1\right)}>a_{1}^{\left(s+1\right)}a_{2}^{\left(s+1\right)},\\ a_{3}^{\left(s+1\right)}>a_{1}^{\left(s+1\right)},\\ a_{1}^{\left(s+1\right)}+a_{3}^{\left(s+1\right)}\geq a_{2}^{\left(s+1\right)}+a_{4}^{\left(s+1\right)}>0; \end{array}$$

case 4. $g_{10}^{\left(s+1\right)}=g_{11}^{\left(s+1\right)}=\frac{a_{3}^{\left(s+1\right)}+a_{4}^{\left(s+1\right)}}{2n}$, $g_{01}^{\left(s+1\right)}=g_{00}^{\left(s+1\right)}=\frac{a_{1}^{\left(s+1\right)}+a_{2}^{\left(s+1\right)}}{2n}$, if

$$\{\begin{array}{l} a_{2}^{\left(s+1\right)}a_{4}^{\left(s+1\right)}>a_{1}^{\left(s+1\right)}a_{3}^{\left(s+1\right)},\\ a_{2}^{\left(s+1\right)}>a_{1}^{\left(s+1\right)},\\ a_{1}^{\left(s+1\right)}+a_{2}^{\left(s+1\right)}\geq a_{3}^{\left(s+1\right)}+a_{4}^{\left(s+1\right)}>0; \end{array}$$

case 5. $g_{01}^{\left(s+1\right)}=g_{11}^{\left(s+1\right)}=\frac{a_{2}^{\left(s+1\right)}+a_{4}^{\left(s+1\right)}}{2n}$, $g_{10}^{\left(s+1\right)}=\frac{a_{3}^{\left(s+1\right)}}{n}$, $g_{00}^{\left(s+1\right)}=\frac{a_{1}^{\left(s+1\right)}}{n}$, if

$$\{\begin{array}{l} a_{4}^{\left(s+1\right)}>a_{2}^{\left(s+1\right)},\\ 2a_{3}^{\left(s+1\right)}\geq a_{2}^{\left(s+1\right)}+a_{4}^{\left(s+1\right)}>0,\\ a_{2}^{\left(s+1\right)}+2a_{3}^{\left(s+1\right)}+a_{4}^{\left(s+1\right)}\leq n; \end{array}$$

case 6. $g_{10}^{\left(s+1\right)}=g_{11}^{\left(s+1\right)}=\frac{a_{3}^{\left(s+1\right)}+a_{4}^{\left(s+1\right)}}{2n}$, $g_{01}^{\left(s+1\right)}=\frac{a_{2}^{\left(s+1\right)}}{n}$, $g_{00}^{\left(s+1\right)}=\frac{a_{1}^{\left(s+1\right)}}{n}$, if

$$\{\begin{array}{l} a_{4}^{\left(s+1\right)}>a_{3}^{\left(s+1\right)},\\ 2a_{2}^{\left(s+1\right)}\geq a_{3}^{\left(s+1\right)}+a_{4}^{\left(s+1\right)}>0,\\ 2a_{2}^{\left(s+1\right)}+a_{3}^{\left(s+1\right)}+a_{4}^{\left(s+1\right)}\leq n; \end{array}$$

case 7. $g_{01}^{\left(s+1\right)}=\frac{a_{2}^{\left(s+1\right)}}{2(a_{2}^{\left(s+1\right)}+a_{3}^{\left(s+1\right)})}$, $g_{10}^{\left(s+1\right)}=\frac{a_{3}^{\left(s+1\right)}}{2(a_{2}^{\left(s+1\right)}+a_{3}^{\left(s+1\right)})}$, $g_{11}^{\left(s+1\right)}=\frac{a_{4}^{\left(s+1\right)}}{2(a_{1}^{\left(s+1\right)}+a_{4}^{\left(s+1\right)})}$, $g_{00}^{\left(s+1\right)}=\frac{a_{1}^{\left(s+1\right)}}{2(a_{1}^{\left(s+1\right)}+a_{4}^{\left(s+1\right)})}$, if

$$\{\begin{array}{l} a_{2}^{\left(s+1\right)}+a_{3}^{\left(s+1\right)}>a_{1}^{\left(s+1\right)}+a_{4}^{\left(s+1\right)},\\ a_{4}^{\left(s+1\right)}>0,\\ a_{1}^{\left(s+1\right)}a_{2}^{\left(s+1\right)}>a_{3}^{\left(s+1\right)}a_{4}^{\left(s+1\right)},\\ a_{1}^{\left(s+1\right)}a_{3}^{\left(s+1\right)}>a_{2}^{\left(s+1\right)}a_{4}^{\left(s+1\right)}. \end{array}$$

The above procedure is iteratively carried out until convergence. Then the restricted MLE ${\hat{\theta }}^{R}$ of *θ *in terms of the restricted MLE ${\hat{g}}^{R}$ can be obtained correspondingly by equations (1).

Compared to the general EM algorithm, the M-step of the REM is a little more complex. It needs some necessary discrimination, then **g**^(*s*+1) ^can be obtained based on **a**^(*s*+1)^. Note that **g**^(*s*+1) ^has the closed-form solution, so it will largely improve the computational efficiency of the parameters. The restricted EM algorithm is convergent, and the restricted MLE ${\hat{\theta }}^{R}$ from the proposed restricted EM algorithm is a consistent estimator of the parameter *θ*.

### Case for more offspring

It is an important fact that more offspring in each family will provide more information in linkage analysis, therefore, and we need to extend the REM algorithm to cases of multiple offspring (sibship) in each family.

We develop a strategy for estimating the two-locus recombination fractions for this case, and the proposed REM algorithm works as a unified method. Taking three-offspring case as an example, we group the observed families into 5 classes according to linkage analysis regulation, with the observed data {*n*_*k*_, *k *= 1,⋯, 5}. After data augmentation, we obtain complete data {*n*_*kl*_, *k *= 1, 2, 3, 4, 5, *l *= 1, 2, 3, 4}. Furthermore, the conditional expectation of the complete-data log-likelihood is

$$Q(g|g^{\left(s\right)},\{n_{k}\})=3[b_{1}^{\left(s+1\right)}ln(1-g_{01}-g_{10}-g_{11})+b_{2}^{\left(s+1\right)}ln(g_{01})+b_{3}^{\left(s+1\right)}ln(g_{10})+b_{4}^{\left(s+1\right)}ln(g_{11})],$$

where $b_{i}^{\left(s+1\right)}$'s have similar expressions with $a_{i}^{\left(s+1\right)}$'s given previously. Then the other steps of the REM are the same as those for the case of two offspring, except replacing $a_{i}^{\left(s+1\right)}$'s by $b_{i}^{\left(s+1\right)}$'s. More offspring's cases are analogous completely. It is helpful to construct and analyze a linkage map using this kind of family data.

### Case for unequal prior probabilities of linkage phases

Affected by many factors (e.g., linkage disequilibrium), each phase of a triply heterozygous parent's genotype may in fact not occur with equal prior probability, but the proposed REM can also be applied to the case of unequal phase probability as a unified method. Let each phase occur with probability *h*_*i *_(*i *= 1, 2, 3, 4), where *h*_*i *_is any fixed number that satisfying 0 ≤ *h*_*i *_≤ 1, and $\sum_{i=1}^{4}h_{i}=1$. In this case, two-offspring family data needs to be grouped into 10 different phenotype classes according to linkage analysis regulation (see Table 3), and we can obtain the observed data {*n*_*k*_, *k *= 1, 2,⋯, 10}. Then we augment the observed data {*n*_*k*_, *k *= 1, 2,⋯, 10} by latent variables {*n*_*kl*_, *k *= 1, 2,⋯, 10, *l *= 1, 2, 3, 4} with corresponding probabilities {*p*_*kl*_, *k *= 1, 2,⋯, 10, *l *= 1, 2, 3, 4}. The major difference from the procedure of the REM for *h*_*i *_= 1/4 (*i *= 1, 2, 3, 4) lies in the expression of conditional expectation for each *n*_*kl *_(*k *= 1, 2,⋯, 10, *l *= 1, 2, 3, 4). Take *n*_11 _as an example, $E(n_{11}|g^{\left(s\right)},n_{1})=n_{1}\frac{h_{1}{\left(g_{00}^{\left(s\right)}\right)}^{2}}{p_{1}^{\left(s\right)}}$, where *h*_1 _is the assigned prior probability of phase I. Repeating the similar procedure given in the REM for *h*_*i *_= 1/4 (*i *= 1, 2, 3, 4), we find that the conditional expectation of the log-likelihood of the complete data still has the form of (4), and only the expressions of the components of **a**^(*s*+1) ^are more complex than those given previously. Using the REM algorithm, we can obtain the restricted MLEs of the two-locus recombination fractions easily.

**Table 3:** Phenotype classification when each linkage phase occur with probability h_i_

*k*	(*i*, *j*)^*a*^	*p*_*k*_
1	(1,1), (8,8), (1,8)	$h_{1}g_{00}^{2}+h_{2}g_{01}^{2}+h_{3}g_{11}^{2}+h_{4}g_{10}^{2}$
2	(2,2), (7,7), (2,7)	$h_{1}g_{01}^{2}+h_{2}g_{00}^{2}+h_{3}g_{10}^{2}+h_{4}g_{11}^{2}$
3	(3,3), (6,6), (3,6)	$h_{1}g_{11}^{2}+h_{2}g_{10}^{2}+h_{3}g_{00}^{2}+h_{4}g_{01}^{2}$
4	(4,4), (5,5), (4,5)	$h_{1}g_{10}^{2}+h_{2}g_{11}^{2}+h_{3}g_{01}^{2}+h_{4}g_{00}^{2}$
5	(1,2), (1,7), (2,8), (7,8)	2((*h*_1 _+ *h*_2_)*g*_00_*g*_01 _+ (*h*_3 _+ *h*_4_)*g*_10_*g*_11_)
6	(3,4), (3,5), (4,6), (5,6)	2((*h*_1 _+ *h*_2_)*g*_10_*g*_11 _+ (*h*_3 _+ *h*_4_)*g*_00_*g*_01_)
7	(2,3), (2,6), (3,7), (6,7)	2((*h*_1 _+ *h*_4_)*g*_01_*g*_11 _+ (*h*_2 _+ *h*_3_)*g*_00_*g*_10_)
8	(1,4), (1,5), (4,8), (5,8)	2((*h*_1 _+ *h*_4_)*g*_00_*g*_10 _+ (*h*_2 _+ *h*_3_)*g*_01_*g*_11_)
9	(1,3), (1,6), (3,8), (6,8)	2((*h*_1 _+ *h*_3_)*g*_00_*g*_11 _+ (*h*_2 _+ *h*_4_)*g*_01_*g*_10_)
10	(2,4), (2,5), (4,7), (5,7)	2((*h*_1 _+ *h*_3_)*g*_01_*g*_10 _+ (*h*_2 _+ *h*_4_)*g*_00_*g*_11_)
		
Total		1

^*a*^(*i*, *j*): see Table 2 for the explanation.

### Simulation methods

We conduct two simulation studies to evaluate the performance and robustness of the proposed REM. In the simulations, we simulate two-offspring family data.

#### Comparing the REM and the unrestricted method

Let *θ*_0 _= (*θ*_*AB*_, *θ*_*BC*_, *θ*_*AC*_) denote the true value of the recombination fraction. In genetics, loci A and B are said to be closely linked when 0 ≤ *θ*_*AB *_≤ 0.1, moderately linked when 0.1 ≤ *θ*_*AB *_≤ 0.2, and loosely linked when 0.2 ≤ *θ*_*AB *_≤ 0.5. To show the advantage of the REM algorithm, we consider six scenarios according to the different combinations of linkage states of loci AB and loci BC: CC, CM, CL, MM, ML, and LL, where C, M, and L denotes close, moderate, and loose linkage, respectively. In each scenario, *θ*_*AB *_and *θ*_*BC *_are respectively taken as 0.05, 0.15, and 0.35 for close, moderate, and loose linkage. *θ*_*AC *_is taken as three equally spaced values which all guarantee that (*θ*_*AB*_, *θ*_*BC*_, *θ*_*AC*_) satisfies the natural restriction (2), and the smaller value and the larger one are near the boundary of the region composed by restriction (2), and the moderate one is inside the region. Since the triply homozygous parent only produces haplotype *abc *in triple backcross family, we can only consider the sampling from the heterozygous parent. For demonstrate purpose, we give the process of generating data for each *θ*_0 _in detail:

1. According to equal probability 1/4, We randomly assign a linkage phase of the heterozygous parent in one family.

2. Generate two haplotypes of two offspring from the heterozygous parent in the family according to the conditional probabilities given in Table 1. The haplotype pair (or the family) is easily classified into one of the four classes in Table 2.

3. Repeat step 1 and 2 for *n *= 300 times, then data {*n*_*k*_} for *n *simulated families can be obtained.

In each scenario of our simulations, for each *θ*_0_, we calculate ${\hat{\theta }}^{U}$ and ${\hat{\theta }}^{R}$ by the unrestricted method and the REM, respectively. Repeating the whole process for *M *= 1000 times, we obtain the averages of ${\hat{\theta }}^{U}$ and ${\hat{\theta }}^{R}$ over 1000 replicates by the two methods (see Table 4). As expected, the averages of ${\hat{\theta }}^{R}$ over 1000 replicates agree better with *θ*_0 _than the averages of ${\hat{\theta }}^{U}$.

**Table 4:** The averages of estimates over 1000 replicates for 300 two-offspring families by unrestricted method and the REM

	Parameters			REM			Unrestricted Method		
			
Scenario^*a*^	*θ*_*AB*_	*θ*_*BC*_	*θ*_*AC*_	${\hat{\theta }}_{AB}^{R}$	${\hat{\theta }}_{BC}^{R}$	${\hat{\theta }}_{AC}^{R}$	${\hat{\theta }}_{AB}^{U}$	${\hat{\theta }}_{BC}^{U}$	${\hat{\theta }}_{AC}^{U}$
CC	0.05	0.05	0.06	0.0495	0.0497	0.0604	0.0499	0.0501	0.0597
			0.075	0.0498	0.0500	0.0751	0.0499	0.0500	0.0752
			0.09	0.0500	0.0500	0.0903	0.0496	0.0499	0.0903
									
CM	0.05	0.15	0.16	0.0502	0.1486	0.1607	0.0502	0.1494	0.1602
			0.175	0.0502	0.1495	0.1742	0.0502	0.1495	0.1745
			0.19	0.0497	0.1508	0.1898	0.0497	0.1509	0.1906
									
CL	0.05	0.35	0.36	0.0496	0.3531	0.3685	0.0496	0.3300	0.3711
			0.375	0.0502	0.3532	0.3777	0.0502	0.3287	0.3837
			0.39	0.0498	0.3534	0.3939	0.0499	0.3344	0.3990
									
MM	0.15	0.15	0.16	0.1487	0.1489	0.1643	0.1504	0.1507	0.1611
			0.225	0.1503	0.1500	0.2248	0.1503	0.1501	0.2252
			0.29	0.1497	0.1508	0.2887	0.1498	0.1509	0.2923
									
ML	0.15	0.35	0.36	0.1505	0.3494	0.3745	0.1505	0.3247	0.3737
			0.425	0.1505	0.3533	0.4274	0.1507	0.3254	0.4310
			0.49	0.1498	0.3481	0.4499	0.1503	0.3331	0.4535
									
LL	0.35	0.35	0.36	0.3443	0.3470	0.3601	0.3573	0.3312	0.3675
			0.425	0.3525	0.3517	0.4305	0.3582	0.3315	0.4272
			0.49	0.3531	0.3524	0.4554	0.3582	0.3255	0.4505

^*a*^Scenario: six combinations of linkage states of loci AB and loci BC (C: close linkage; M: moderate linkage; L: loose linkage).

To better show the performance of the REM, we mainly use the following three measures of accuracy to compare ${\hat{\theta }}^{U}$ and ${\hat{\theta }}^{R}$:

1. The number, denoted by *KK*, for which the unrestricted methods give unreasonable estimates based on 1000 replicates.

2. The standard derivations (SDs) of the estimate ${\hat{\theta }}_{i}^{R}$; the ratio of SDs of two kinds of estimates being $\text{rSD}=\text{SD}({\hat{\theta }}_{i}^{U})/\text{SD}({\hat{\theta }}_{i}^{R})$, *i *= *AB*, *BC*, *AC*.

3. The mean absolute error (MAE) of the estimate ${\hat{\theta }}^{R}$, where $\text{MAE}=\sum_{l=1}^{1000}(|{\hat{\theta }}_{ABl}^{R}-\theta_{AB}|+|{\hat{\theta }}_{BCl}^{R}-\theta_{BC}|+|{\hat{\theta }}_{ACl}^{R}-\theta_{AC}|)/3000$; the ratio of MAEs being rMAE = MAE(${\hat{\theta }}^{U}$)/MAE(${\hat{\theta }}^{R}$).

The comparisons of estimations of two-locus recombination fraction by the unrestricted method and the REM are listed in Table 5. In each scenario, the unrestricted method gives lots of unreasonable results, i.e., the estimates do not satisfy the natural restriction (2), whereas the estimates obtained by the proposed REM all satisfy the restriction. The number *KK *of unreasonable estimates is larger when the true value *θ*_0 _is near the boundary of the restriction region (2), which corresponds to the larger or smaller true values of *θ*_*AC*_, and *KK *is somewhat smaller when *θ*_0 _is inside the region, which corresponds to the moderate values of *θ*_*AC*_. In the former situation the resulting ${\hat{\theta }}^{U}$ could be obtained in the whole parameter space but not in the restriction region (2). When *θ*_0 _is near the boundary of the restriction region (2), ${\hat{\theta }}^{U}$ is liable to be near the boundary of the region and hence likely to lie outside the boundary. However the proposed method can guarantee that ${\hat{\theta }}^{R}$ must be inside the restriction region at any time.

**Table 5:** Comparison of estimation of two-locus recombination fraction for 300 two-offspring families by the unrestricted method and the REM

	Parameters			SD			rSD^*b*^					
						
Scenario^*a*^	*θ*_*AB*_	*θ*_*BC*_	*θ*_*AC*_	${\hat{\theta }}_{AB}^{R}$	${\hat{\theta }}_{BC}^{R}$	${\hat{\theta }}_{AC}^{R}$	${\hat{\theta }}_{AB}^{U}$	${\hat{\theta }}_{BC}^{U}$	${\hat{\theta }}_{AC}^{U}$	MAE^*c*^	rMAE^*d*^	*KK*^*e*^
CC	0.05	0.05	0.06	0.0089	0.0088	0.0095	1.0606	1.0790	1.1170	0.0072	1.0434	220
			0.075	0.0090	0.0092	0.0114	1.0029	1.0033	1.0187	0.0078	1.0043	6
			0.09	0.0091	0.0093	0.0127	1.0020	1.0022	1.0274	0.0083	1.0062	84
												
CM	0.05	0.15	0.16	0.0093	0.0180	0.0177	1.0007	1.0603	1.0560	0.0119	1.0223	183
			0.175	0.0091	0.0182	0.0195	1.0007	1.0168	1.0562	0.0124	1.0140	34
			0.19	0.0094	0.0183	0.0209	1.0012	1.0122	1.1352	0.0128	1.0299	197
												
CL	0.05	0.35	0.36	0.0095	0.0463	0.0481	1.0008	4.2411	1.4711	0.0272	1.2941	502
			0.375	0.0090	0.0464	0.0482	1.0009	4.2875	1.6143	0.0272	1.3343	487
			0.39	0.0093	0.0445	0.0467	1.0006	3.8670	1.7417	0.0267	1.3462	518
												
MM	0.15	0.15	0.16	0.0156	0.0168	0.0168	1.2658	1.1893	1.2115	0.0131	1.0956	451
			0.225	0.0181	0.0176	0.0239	1.0078	1.0080	1.0863	0.0159	1.0174	1
			0.29	0.0174	0.0187	0.0261	1.0117	1.0098	1.6503	0.0166	1.1165	343
												
ML	0.15	0.35	0.36	0.0177	0.0452	0.0514	1.0024	5.1260	1.4805	0.0298	1.3264	419
			0.425	0.0179	0.0459	0.0504	1.0071	5.0395	1.5690	0.0311	1.3790	297
			0.49	0.0179	0.0410	0.0584	1.0167	4.9795	1.3348	0.0303	1.2595	304
												
LL	0.35	0.35	0.36	0.0390	0.0373	0.0454	2.0082	6.4504	1.5975	0.0319	1.4403	604
			0.425	0.0454	0.0436	0.0498	1.4563	4.3277	1.5612	0.0378	1.2931	278
			0.49	0.0460	0.0465	0.0577	1.3683	4.5456	1.4778	0.0375	1.3018	216

^*a*^Scenario: see Table 4 for the explanation;

^*b*^$\text{rSD}=\text{SD}({\hat{\theta }}_{i}^{U})/\text{SD}({\hat{\theta }}_{i}^{R})$, *i *= *AB*, *BC*, *AC*;

^*c*^$\text{MAE}=\sum_{l=1}^{1000}(|{\hat{\theta }}_{ABl}^{R}-\theta_{AB}|+|{\hat{\theta }}_{BCl}^{R}-\theta_{BC}|+|{\hat{\theta }}_{ACl}^{R}-\theta_{AC}|)/3000$: the mean absolute error of ${\hat{\theta }}^{R}$;

^*d*^rMAE = MAE(${\hat{\theta }}^{U}$)/MAE(${\hat{\theta }}^{R}$);

^*e*^*KK*: number for which the unrestricted method gives unreasonable estimates based on all 1000 replicates.

It is clear to see that our REM outperforms the unrestricted method for estimating two-locus recombination fractions in each simulated scenario. The estimates obtained by the REM have smaller SDs than the unrestricted method, which is more obvious especially at least one of the intervals of AB and BC is loosely linked. This suggests that the accuracy of estimates by the REM is more higher than by the unrestricted method, and that the natural restriction (2) should be taken into account in estimating, otherwise it would have significant impact on the accuracy on practical inference. Compared to ${\hat{\theta }}^{U}$, ${\hat{\theta }}^{R}$ is closer to the true value *θ*_0 _(rMAE > 1 for all groups in Table 5).

It also can be seen that the proposed REM is a robust algorithm. The REM can still give better results than the unrestricted method in each scenario even when *KK *is small (e.g., 1).

#### Evaluating the effect of interference to estimates

Interference refers to the phenomenon that crossovers in nearby intervals along a chromosome do not occur independently. Let *I *denote the value of interference. According the definition of interference in Strickberger [21], we have $I=1-\frac{g_{11}}{\theta_{AB}\theta_{BC}}$. To better evaluate the effect of interference to the two kinds of estimations, we consider three scenarios: positive, null and negative interferences. In each scenario, we choose equal *θ*_*AC *_and different *θ*_*AB *_and *θ*_*BC *_corresponding to different interference values (see Table 6). For each scenario, we also simulate 300-family data, and the REM and the unrestricted method are applied to the simulated data, respectively. The whole process is repeated for 1000 times to compute the measures of accuracy given previously. The simulation results listed in Table 6 firstly show that the values of *KK *are very large when there exists positive (or negative) interference, and the values are small when there is no interference, while the REM gives reasonable estimates at any time. That is to say the estimating results by the unrestricted method are much affected by the interference, but the results by our REM is less affected. Secondly, the less fluctuations of SD(${\hat{\theta }}_{AC}^{R}$) in scenario 1 (or 3) also validate that the REM is less affected by interference. Finally, the REM outperforms the unrestricted method in each scenario (rSD > 1, rMAE > 1), especially, when negative interference is present.

**Table 6:** Evaluation of the effect of interference to estimates of recombination fractions

Scenario	*θ*_*AB*_	*θ*_*BC*_	*θ*_*AC*_	*I*^*a*^	SD(${\hat{\theta }}_{AC}^{R}$)	*rSD*^*b*^	MAE^*c*^	rMAE^*d*^	*KK*^*e*^
1	0.031	0.060	0.09	0.7312	0.0124	1.0484	0.0078	1.0128	475
	0.035	0.056	0.09	0.7449	0.0124	1.0323	0.0080	1.0125	487
	0.039	0.052	0.09	0.7535	0.0120	1.0250	0.0078	1.0128	472
									
2	0.081	0.1301	0.19	0	0.0205	1.0537	0.0131	1.0229	64
	0.085	0.1265	0.19	0	0.0196	1.0765	0.0127	1.0315	72
	0.089	0.1229	0.19	0	0.0198	1.0505	0.0127	1.0236	48
									
3	0.151	0.359	0.39	-0.1068	0.0553	1.1971	0.0327	1.3945	354
	0.155	0.355	0.39	-0.0904	0.0549	1.2095	0.0321	1.2928	323
	0.159	0.351	0.39	-0.0751	0.0552	1.2156	0.0324	1.3025	300

^*a*^*I*: value of interference;

^*b*^rSD, ^*c *^MAE, ^*d *^rMAE and ^*e*^*KK*: see Table 5 for the explanations.

In addition, we find that the restricted EM estimate is little changed when different starting values are taken. These above results indicate that the use of the REM can yield better performance than the current unrestricted method.

### A worked example

We applied our proposed method to a real data set from published literature [22]. The data set comprised of 134 individuals from a backcross of mice. Here we consider the three ordinal marker loci D2Mit365, D2Mit272 and D2Mit456 on the linkage map of chromosome 2, and we still use A, B and C to denote the three loci. According to the genotypes given in the data set, we record a haplotype code of each individual, where the haplotype is from the heterozygous parent. Two individuals are randomly grouped into one family, and we consider they are really from that family, where the treatment will not affect linkage information, because all offspring's genotypes are independent conditional on the genotypes of all parents for the data. Then we obtain *n *= 67 two-offspring families, and *n*_1 _= 21, *n*_2 _= 17, *n*_3 _= 14 and *n*_4 _= 15 by the classification given in Table 2. We used the proposed REM and the unrestricted method to estimate the recombination fractions based on (*n*_1_, *n*_2_, *n*_3_, *n*_4_). The MLEs of the recombination fractions are ${\hat{\theta }}_{AB}^{R}$ = 0.3166, ${\hat{\theta }}_{BC}^{R}$ = 0.3738 and ${\hat{\theta }}_{AC}^{R}$ = 0.3738; and ${\hat{\theta }}_{AB}^{U}$ = 0.3167, ${\hat{\theta }}_{BC}^{U}$ = 0.3942 and ${\hat{\theta }}_{AC}^{U}$ = 0.3634, respectively. Obviously, the unrestricted estimates do not satisfy the second one of the natural restriction (2), and thus estimates contradict with the true order of the three markers on the linkage map of chromosome 2 [22]. According to our simulation and practical experience, the accuracy of estimation by the REM will improve by increasing sample size or by using the unrestricted estimates as initial values.

## Discussion

We developed a restricted EM algorithm to calculate numerically the MLEs of two-locus recombination fractions that initially studied by Ott [3]. The method in Ott [3] may not always provide the parameter estimates satisfying the natural restriction (2), since the approach does not take the inequality restrictions into account. Our method can deal with this problem, and the real data were handled very well with the proposed method.

The performance of the REM is also illustrated using simulated data. Our simulation shows that the unrestricted method gives some unreasonable estimate results in each scenario, and thus such estimates may not provide correct interpretation of the recombination phenomenon in practice. The major advantage of the REM is its robustness and efficiency. The REM can give better results even when the number for which the unrestricted method gives unreasonable estimate results is small (e.g., *KK *= 1), and our estimates are more precise than those obtained by the unrestricted method. Moreover, the REM is less affected by interference, and the estimate of parameter g in M-step having the closed-form solution largely improves the computational efficiency of the parameter.

On the other hand, noticing the important fact that more offspring in each family can really provide more information in linkage analysis, we develops a strategy for estimating the two-locus recombination fractions when each observed family has more offspring, and the proposed REM algorithm works as a unified method. In practice, the method developed by Lu *et al*. [10] can be first adopted to obtain the estimates of probabilities *h*_*i*_'s of linkage phases when considering multiple offspring, then the REM is used to obtain the restricted MLEs of recombination fractions, which may improve the estimation precision. It is helpful to construct and analyze a linkage map using this kind of family data.

Recent research in genetics has shown that statistical inference about the two-locus recombination fraction offers an effective approach for constructing and analyzing a linkage map between the genetic marker and the genetic disorders. Reasonable estimates of the recombination fractions are important in gene mapping, especially in interval mapping [23-26]. Only the reasonable estimate result may identify the actual genes responsible for some trait, and it is feasible to embed the REM into interval mapping to improve the efficiency of mapping.

It is noticed that our analysis is focused on three biallelic loci. The above constrained parameter problem may become complicated if the number of loci is more than three, or some markers may have more alleles than others, for example, in outcrossing plant species. When the number of loci is more than three, we suggest that every three adjacent loci are subject to three-point analysis. We can obtain two different estimates of the recombination fraction for the same marker interval, and a better way to combine these estimates is to take a weighted mean. More alleles for each markers mean more possible linkage phases [10], which bring some difficulty to linkage analysis, however, the idea of considering the natural restriction (2) on recombination fractions should also be emphasized. Further investigation in this area is warranted.

## Appendix

### The Kuhn-Tucker Theorem [19,20]

Suppose that θ* is a solution of

*Max f*(*θ*) subject to *f*_1_(*θ*) ≥ 0,⋯, *f*_*m*_(*θ*) ≥ 0,

where *f*, *f*_1_,⋯, *f*_*m*_: *R*^*N *^→ *R *are *C*^1 ^functions. Then the following conditions hold:

(1) $\frac{\partial }{\partial \theta_{i}}f(\theta^{*})+\sum_{j=1}^{m}\lambda_{j}\frac{\partial }{\theta_{i}}f_{j}(\theta^{*})=0$, *i *= 1,⋯, *N*;

(2) *λ*_*j*_*f*_*j*_(θ*) = 0, *j *= 1,⋯, *m*;

(3) *f*_*j*_(θ*) ≥ 0, *j *= 1,⋯, *m*;

(4) *λ*_*j *_≥ 0, *j *= 1,⋯, *m*,

where (*λ*_1_,⋯, *λ*_*m*_) are Lagrangian multipliers. The four conditions are called Kuhn-Tucker conditions. Specially, if *f*(*θ*) is strictly concave and the set {*θ*: *f*_1_(*θ*) ≥ 0,⋯, *f*_*m*_(*θ*) ≥ 0} is convex, the Kuhn-Tucker conditions are also sufficient, and the solution θ* is unique.

### Solving equation (5) when ${\tilde{g}}^{\left(s+1\right)}$ does not satisfy restriction (3)

Because *Q*(**g**|**g**^(*s*)^, {*n*_*k*_}) is a strictly concave function and the restriction region (3) is a convex set, there must be a unique solution $ˇg^{\left(s+1\right)}$ to equation (5) by the Kuhn-Tucker Theorem. The Lagrangian is

*L*(**g**, *λ*) = *Q*(**g**|**g**^(*s*)^, {*n*_*k*_}) + *λ*_1_(*g*_01 _- *g*_11_) + *λ*_2_(*g*_10 _- *g*_11_) + *λ*_3_*g*_11 _+ *λ*_4_(1/2 - *g*_01 _- *g*_10_),

where *λ *= (*λ*_1_, *λ*_2_, *λ*_3_, *λ*_4_), and *λ*_*i*_'s are Lagrangian multipliers. Then $ˇg^{\left(s+1\right)}=(ˇg_{10}^{\left(s+1\right)},ˇg_{01}^{\left(s+1\right)},ˇg_{11}^{\left(s+1\right)})$ is a unique solution to

$$\{\begin{array}{l} -\frac{a_{1}^{\left(s\right)}}{g_{00}}+\frac{a_{3}^{\left(s\right)}}{g_{10}}+\lambda_{2}-\lambda_{4}=0,\\ -\frac{a_{1}^{\left(s\right)}}{g_{00}}+\frac{a_{2}^{\left(s\right)}}{g_{01}}+\lambda_{1}-\lambda_{4}=0,\\ -\frac{a_{1}^{\left(s\right)}}{g_{00}}+\frac{a_{4}^{\left(s\right)}}{g_{11}}-\lambda_{1}-\lambda_{2}+\lambda_{3}=0,\\ \lambda_{1}(g_{01}-g_{11})=0,\\ \lambda_{2}(g_{10}-g_{11})=0,\\ \lambda_{3}g_{11}=0,\\ \lambda_{4}(1/2-g_{01}-g_{10})=0,\\ g_{01}\geq g_{11},\\ g_{10}\geq g_{11},\\ g_{11}\geq 0,\\ 1/2\geq g_{01}+g_{10},\\ \lambda_{i}\geq 0,i=1,2,3,4. \end{array}$$

To solve the above equations, we need to consider all possible cases for *λ*_*i *_= 0 or *λ*_*i *_> 0, *i *= 1, 2, 3, 4. There are totally seven possible solutions for the above equations which were just given in the previous REM algorithm.

## Authors' contributions

YZ derived the genetic and statistical model and wrote computer programs. NZS and WKF provided insightful comments to the presentation. JG conceived of ideas and algorithm. All authors read and approved the final manuscript.
